# Whole-liver histogram and texture analysis on T1 maps improves the risk stratification of advanced fibrosis in NAFLD

**DOI:** 10.1007/s00330-020-07235-4

**Published:** 2020-09-08

**Authors:** Xinxin Xu, Hong Zhu, Ruokun Li, Huimin Lin, Robert Grimm, Caixia Fu, Fuhua Yan

**Affiliations:** 1 Department of Radiology, Ruijin Hospital, Shanghai Jiao Tong University School of Medicine, No. 197 Ruijin Er Road, Shanghai, 200025 China; 2 MR Applications Predevelopment, Siemens Healthcare, Erlangen, Germany; 3 MR Applications Development, Siemens Shenzhen Magnetic Resonance Ltd, Shenzhen, People’s Republic of China

**Keywords:** Fibrosis, NAFLD, Magnetic resonance imaging, Diagnostic imaging

## Abstract

**Objectives:**

To assess whole-liver texture analysis on T1 maps for risk stratification of advanced fibrosis in patients with suspected nonalcoholic fatty liver disease (NAFLD).

**Methods:**

This retrospective study included 53 patients. Histogram and texture parameters (volume, mean, SD, median, 5th percentile, 95th percentile, skewness, kurtosis, diff-entropy, diff-variance, contrast, and entropy) of T1 maps were calculated based on the semi-automatically segmented whole-liver volume. A two-step approach combining the Nonalcoholic Fatty Liver Disease Fibrosis Score (NFS) and Fibrosis-4 Index (FIB-4) with the liver stiffness measurement (LSM) for the risk stratification was used. Univariate analysis was performed to identify significant parameters. Logistic regression models were then run on the significant features. Diagnostic performance was evaluated with receiver operating characteristic (ROC) analysis.

**Results:**

In total, 33 (62%) subjects had a low risk and 20 (38%) subjects had an intermediate-to-high risk of advanced fibrosis. The following significantly different parameters with the best performance were diff-entropy, entropy, and diff-variance, with AUROC 0.837 (95% CI 0.73–0.95), 0.821 (95% CI 0.71–0.94), and 0.807 (95% CI 0.69–0.93). The optimal combination of median, 5th percentile, and diff-entropy as a multivariate model improved the diagnostic performance to diagnose an intermediate-to-high risk of advanced fibrosis with AUROC 0.902(95% CI 0.79–0.97).

**Conclusions:**

Parameters obtained by histogram and texture analysis of T1 maps may be a noninvasive analytical approach for stratifying the risk of advanced fibrosis in NAFLD.

**Key Points:**

• *Variable flip angle (VFA) T1 mapping can be used to acquire 3D T1 maps within a clinically acceptable duration.*

• *Whole-liver histogram and texture parameters on T1 maps in patients with NAFLD can distinguish those with an intermediate-to-high risk of advanced fibrosis.*

• *The multivariate model of combination of texture parameters improved the diagnostic performance for a high risk of advanced fibrosis and clinical parameters offer no added value to the multivariate model.*

**Electronic supplementary material:**

The online version of this article (10.1007/s00330-020-07235-4) contains supplementary material, which is available to authorized users.

## Introduction

Nonalcoholic fatty liver disease (NAFLD) is the most common cause of chronic liver disease and is estimated to affect 25% of the general population in the Asia-Pacific region [1]. The presence of fibrosis, particularly advanced fibrosis, is the most important prognostic factor in NAFLD and is correlated with liver-related outcomes and mortality [2, 3]. Monitoring fibrosis progression and recognizing those individuals at high risk of advanced fibrosis is important because those patients might benefit from a tailored therapeutic strategy [4]. However, biopsy is invasive and problematic for frequent monitoring. Moreover, its interpretation is in part subjective [5]. For these reasons, noninvasive and objective techniques are under investigation, including fibrosis-specific serum markers, ultrasound elastography, magnetic resonance (MR) elastography, and diffusion-weighted MR imaging [6].

Recently, the quantification of T1 relaxation time on parametric maps demonstrated its great potential as a reliable and accurate method for noninvasively monitoring liver fibrosis, because T1 relaxation time increased with fibrosis progression and decreased with regression [7, 8]. The prolonged T1 relaxation time was likely caused by the structural and pathophysiological alterations associated with induced liver fibrogenesis, which is characterized by edema, inflammation, and excess deposition of extracellular matrix (ECM) [9, 10]. The true potential of T1 mapping techniques for the detection of fibrosis in NAFLD remains unclear, because steatosis and iron deposition that are characteristic of NAFLD progression might shorten the T1 time [11] and act as a confounder when only T1 value is used to detect fibrosis.

Texture analysis allows the assessment of the internal organization of a tissue. It also detects tissue changes that are imperceptible to the human eye. Recently, its application to liver fibrosis has been described in the setting of hepatitis B virus (HBV) infection, NAFLD, and in animal models, demonstrating the potential of texture analysis for staging liver fibrosis [12–14].

Thus, the purpose of our study was to assess the diagnostic potential of texture analysis applied to T1 maps for the risk stratification of advanced fibrosis in NAFLD.

## Materials and methods

### Patients

**Fig. 1:**
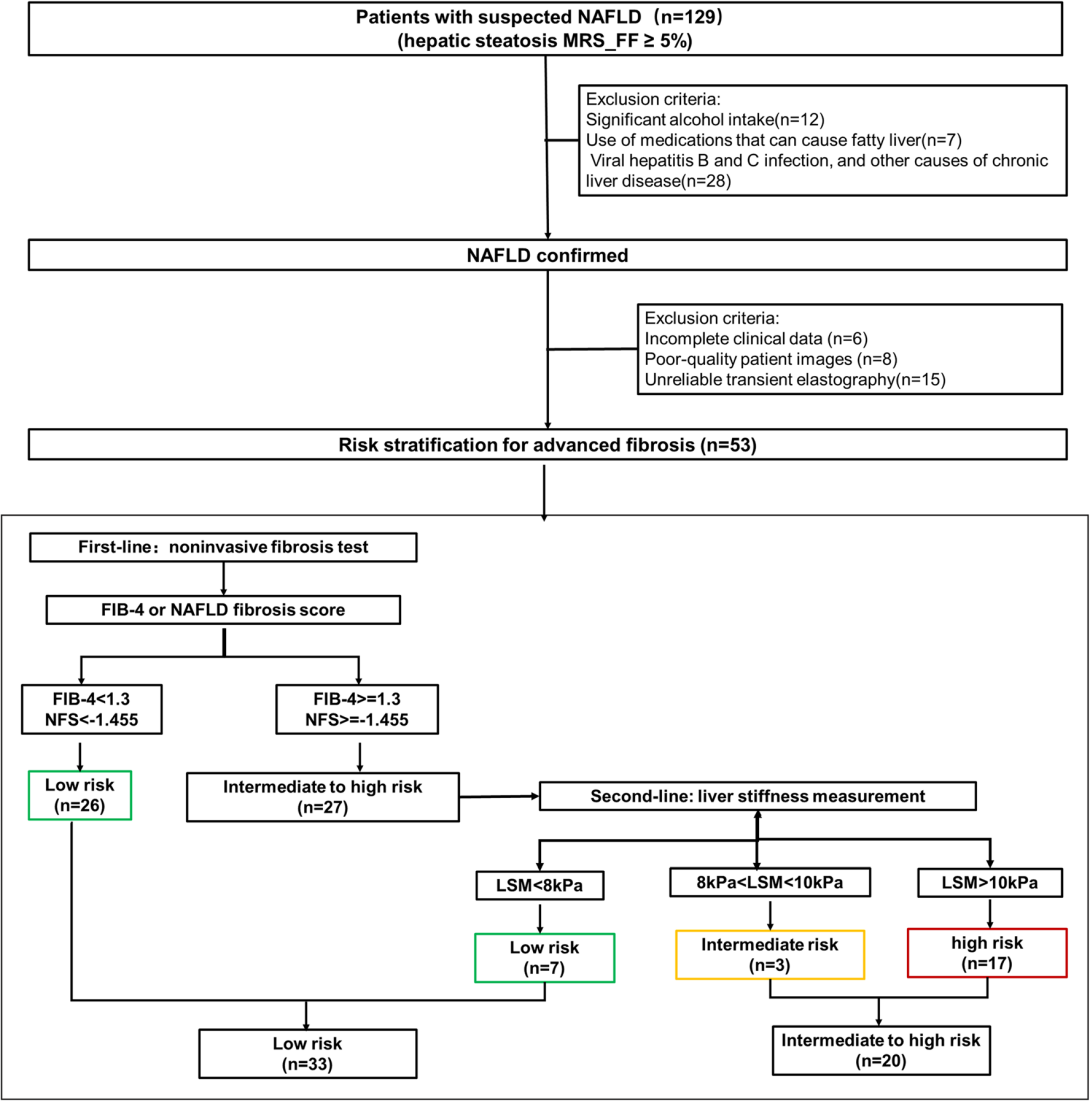
Flowchart of stratifying risk of advanced fibrosis

A series of 129 consecutive patients suspected of having NAFLD (defined as the presence of steatosis on ultrasound or abnormal liver tests (high levels of alanine aminotransferase, aspartate aminotransferase, g-glutamyl transferase in the blood)) who underwent both MRI and MRS examinations in our hospital between August 2018 and July 2019 were enrolled in the study. Liver MRI, transient elastography, and fasting blood samples were completed within 2 weeks. A total of 76 patients were excluded for the following reasons: significant alcohol intake (*n* = 12), use of medications that can cause fatty liver (*n* = 7), viral hepatitis B and C infection, and other causes of chronic liver disease (*n* = 28), incomplete clinical data (*n* = 6), poor-quality images of patients (*n* = 8), and unreliable transient elastography (*n* = 15). Finally, 53 patients with a median age of 46 years (age range, 24–73 years), including 22 (42%) women and 31 (58%) men, were enrolled in this retrospective study (Fig. 1). Our institutional review board approved this retrospective study and waived the requirement for written informed consent.

### MR imaging

MR imaging was performed on a MAGNETOM Aera 1.5T MR system (Siemens Healthcare). The MR examinations included high-speed T2-corrected multiecho single-voxel spectroscopy (HISTO) [15], B1 mapping, and T1 mapping sequences in the transversal plane.

The HISTO sequence for liver fat fraction estimation was executed with the following parameters: repetition time (TR) = 3000 ms; TE = 12/24/36/48/72 ms; averages = 1; bandwidth = 1200 Hz/pixel; voxel size = 3 × 3 × 3 cm^3^; vector size = 1024; and acquisition duration = 15 s in one breath-hold. The ROI was positioned on the right lobe of the liver, avoiding major hepatic vessels. The T2-corrected fat fraction was calculated inline by the scanner based on the multiecho spectroscopy data after acquisition.

B1 mapping using Turbo-FLASH acquisition was executed before T1 mapping, with the following parameters: TR/TE = 4280/2.04 ms; field of view = 380 mm × 309 mm; flip angle = 8°; matrix = 64 × 64; slice thickness = 8 mm; slice distance factor = 100%; number of slices = 18; and duration = 9 s.

The transversal T1 map was obtained using volume interpretation breath-hold examination (VIBE) sequence and the variable flip angle (VFA) method with flip angles of 3° and 15°, which were automatically calculated by the software based on the TR and the estimated target T1 of 1000 ms. The position of the center slice of T1 map was kept consistent with that of the B1 map. Other scan parameters were as follows: TR/TE = 4.61/2.26 ms; field of view = 380 mm × 309 mm; matrix = 179 × 256; slice thickness = 3.5 mm; slices per slab = 72; bandwidth = 350 Hz/pixel; and duration = 19 s. The T1 map was inline generated after the data acquisition. B1 field correction was automatically executed using the interpolated B1 map during the calculation of the T1 mapping.

For the measurement of inter-examination repeatability, five volunteers underwent three consecutive, same-day MR examinations including B1 mapping and T1 mapping. Between examinations, volunteers left the scanner for 5 min and were then repositioned on the scanner table; then, the phased-array coil was reconnected, and the next examination was performed.

### Histogram and texture analysis

Whole-liver histogram and texture analysis of the T1 map were performed with the prototype MR multiparametric analysis software (Siemens Healthcare) by the radiologist (X.X.). The 3D analysis of the T1 map included the following four steps:

1. Data loading: Original images with a flip angle of 15° and the T1 map were loaded onto the software.
2. Seed points drawing: Foreground seed points were manually drawn inside the liver parenchyma which represent regions within the volume of interest (VOI). Background seed points were manually drawn outside the liver parenchyma and inside the liver blood vessels which contain voxels outside the VOI. In axial plane, foreground and background seed points were drawn on the slices which are close to the top, the middle, and the bottom of the liver. In sagittal and coronal plane, seed points were drawn on the slices with the maximum liver cross-section.
3. Segmentation: The whole liver was segmented by the software based on the seed points using a random walker algorithm. The segmentation region (pink) was then checked slice by slice; manual adjustments were performed if the initial segmentation result was not satisfactory. The final 3D-segmented volumes created on the original images with 15° were then automatically propagated to the T1 map.
4. Histogram and texture analyses: The whole-liver histogram and texture analyses on the T1 map were automatically performed by a one-push button. A total of eight histogram-based statistical features and four texture-based features were extracted. The histogram-based features included the volume, mean, standard deviation (SD), median, percentiles (5th and 95th), skewness (defined as a measure of asymmetry of the probability distribution), and kurtosis (measure of the shape of the probability distribution). Texture-based features included entropy (measure of the randomness of the gray levels), contrast (measure of the amount of gray-level variations), difference entropy (diff-entropy, measure of the entropy difference), and difference variance (diff-variance, measure of variation in the difference in gray levels between voxel pairs).

**Fig. 2:**
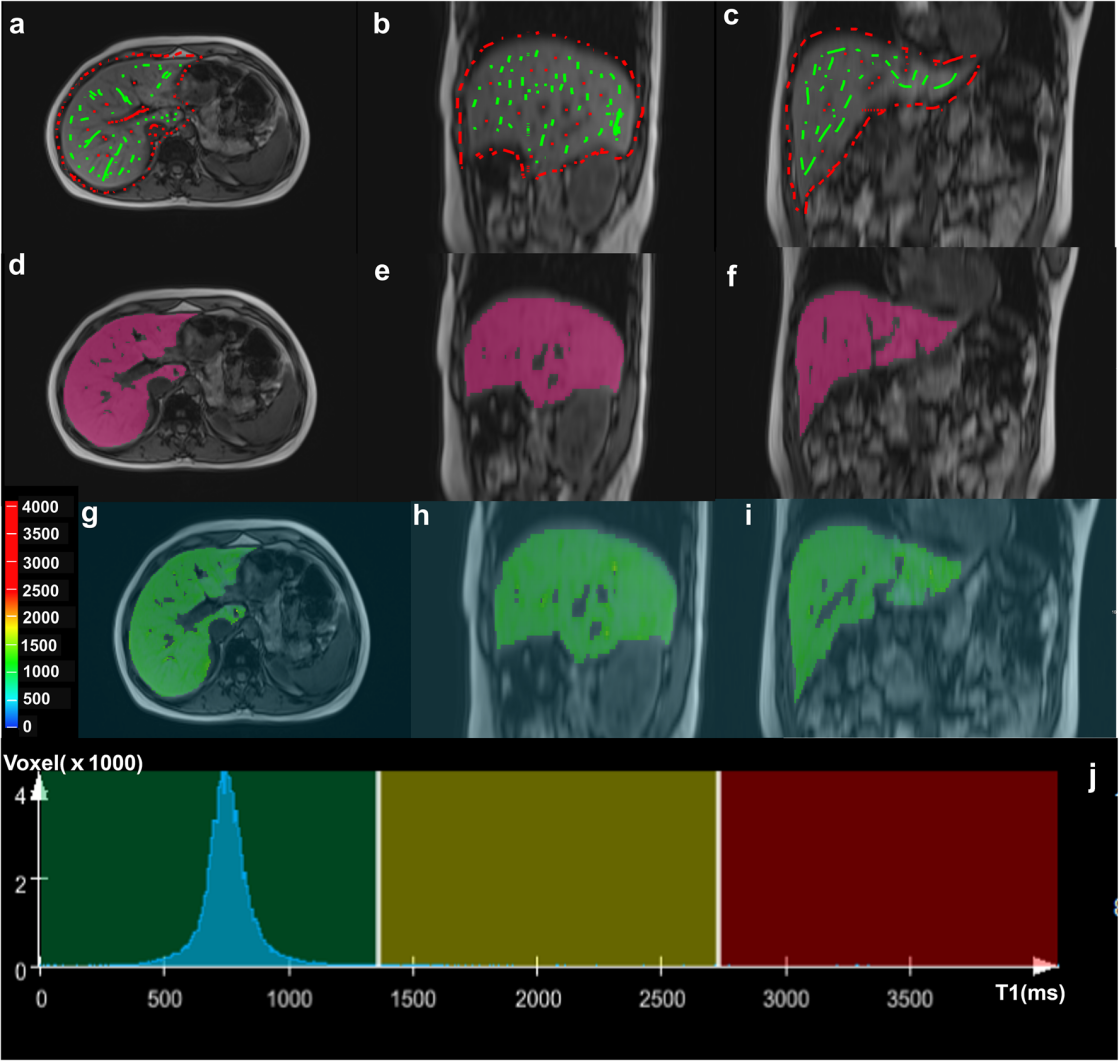
Workflow of the histogram and texture analysis. **a**–**c** Foreground and background seed points were manually drawn inside the liver (green color) and outside the liver/in main vessels (red color) on the three multiplane reconstruction planes of images with a flip angle of 15°. **d**–**f** Whole-liver segmentation created on post-contrast images. **g**–**j** Histograms for the T1 maps

Steps 2, 3, and 4 are illustrated in Fig. 2.

To test the reproducibility of all the histogram and texture parameters, whole-liver histogram and texture analysis were repeated twice after a pause of 2 weeks in a randomly selected subgroup of 20 study participants by the same reader for intra-observer analysis and by another reader (H.M.L., with 6 years of experience in liver MRI) for the inter-observer analysis. Both readers were blinded to the results of the first reading, as well as all the clinical data. Lastly, total time of four steps and the time required for drawing seed points was recorded by both readers.

### Risk stratification of advanced fibrosis

#### Blood fibrosis tests

The Nonalcoholic Fatty Liver Disease Fibrosis Score (NFS) was calculated using the following formula: − 1.675 + 0.037 × age (years) + 0.094 × body mass index (kg/m^2^) + 1.13 × impaired glucose tolerance or diabetes mellitus (yes = 1, no = 0) + 0.99 × aspartate aminotransferase to alanine aminotransferase ratio − 0.013 × platelet (10^9^/L) − 0.66 × albumin (g/dL).

The Fibrosis-4 Index (FIB4) was calculated using the following formula: age (years) × aspartate aminotransferase/[platelet (10^9^/L) × square root (alanine aminotransferase)].

#### Liver stiffness measurement

Liver stiffness measurements were performed using vibration-controlled transient elastography technology (FibroScan device; Echosens), using an M probe.

An examination was considered successful if there were at least 10 valid measurements and were reliable if the IQR/median of the LSM was 30% or less, or if the LSM was less than 7.1 kPa when the interquartile range/median of the LSM was greater than 30% [16].

#### Combining blood fibrosis tests and liver stiffness measurement for risk stratification of advanced fibrosis

A 2-step approach (blood fibrosis test—first-line, LSM—second-line) has recently been proposed in the guidelines of the European Association for the Study of the Liver (EASL) [4, 17, 18].

The approach uses NFS or FIB-4 [18, 19] as the first-line procedure (Fig. 1): Patients with FIB-4 < 1.3 or NFS < − 1.455 were considered to be at low risk of advanced fibrosis; patients with FIB-4 = 1.3 to 3.25 or NFS = − 1.455 to 0.672 were considered to be at intermediate risk, patients with FIB-4 > 3.25 or NFS > 0.672 were considered to be at high risk of having advanced fibrosis.

When the first-line test showed an intermediate or high risk, a second-line evaluation of LSM was performed: an LSM of less than 8 kPa was considered to be low, 8 kPa~10 kPa was considered intermediate, and 10 kPa or greater was considered to be a high risk of having advanced fibrosis.

### Statistical analysis

Intra- and inter-observer agreement in the measurement of all of the T1 map histograms and texture parameters were estimated by calculating the intraclass correlation coefficient (ICC) (0.000–0.200, poor; 0.201–0.400, fair; 0.401–0.600, moderate; 0.601–0.800, good; and 0.801–1.000, excellent). An ICC value greater than 0.80 was considered to indicate excellent agreement. The repeatability of T1 mapping was assessed between three acquisitions. ICCs with 95% confidence intervals were calculated using a two-way mixed model.

All quantitative variables were expressed as means ± standard deviation (SD) and were first tested with the Shapiro-Wilk test for normality analysis. With the univariate analyses, the significant parameter selection was performed using the Student’s *t* test when the data were normally distributed or Mann-Whitney *U* test when not normally distributed. Binary logistic regression analysis with a backward stepwise selection procedure was performed to identify the independent parameter for differentiating the low-risk from the intermediate-to-high-risk group. Multivariate model calibration was assessed with the goodness-of-fit Hosmer-Lemeshow test through a calibration plot. The diagnostic performance of each histogram- and texture-extracted parameter was tested via receiver operating characteristic (ROC) analysis. Cutoff values were established by calculating the maximal Youden index (Youden index = sensitivity + specificity − 1). Intermediate-to-high risk of advanced fibrosis was defined as a positive result. The DeLong test was used to compare the area under the ROC curve (AUC) between the multivariate model and individual parameters and between the multivariate model and noninvasive fibrosis tests. All statistical analyses were performed using SPSS 22.0 (IBM) and MedCalc 15.6.1 (MedCalc). A two-tailed *p* value of < 0.05 indicated statistical significance.

## Results

### Patient clinical characteristics

The flowchart of risk stratification of advanced fibrosis is shown in Fig. 1. As the proportion of subjects at intermediate risk of advanced fibrosis was low (5.7%), we regrouped all patients into either a low-risk group or intermediate-to-high-risk group. Among 53 patients included in our final study population, 33 (62.2%) were in the low-risk group and 20 (37.8%) were in the intermediate-to-high-risk group. The patient characteristics are shown in Table 1. The proportion of patients with diabetes mellitus (DM), aspartate aminotransferase (AST), and triglycerides in the intermediate-to-high-risk group was significantly higher than those in the low-risk group (*p* = 0.008, 0.003, and 0.007, respectively). The platelet count in the low-risk group was significantly higher than that in the intermediate-to-high-risk group (*p* = 0.017). No significant differences were found across groups with respect to age, sex, BMI, or other blood tests, as shown in Table 1 (all *p* > 0.05).

**Table 1:** Patient characteristics

Characteristics	All NAFLD patients (*n* = 53)	Low risk of advanced fibrosis (*n* = 33)	Intermediate-to-high risk of advanced fibrosis (*n* = 20)	*p*
Age (years)	46 ± 15	46 ± 15	43 ± 15	0.551
Male (*n*, %)	31 (58.4)	18 (54.5)	13 (65)	0.458
BMI (kg/m^2^)	26.02 ± 3.35	25.38 ± 2.70	27.07 ± 2.98	0.147
DM (*n*, %)	13 (24.5)	4 (12.1)	9 (45)	0.008*
Bilirubin (μmol/L)	20.25 ± 25.61	21.8 ± 32.1	17.7 ± 6.8	0.409
ALT (IU/L)	77 ± 59	69 ± 62	89 ± 53	0.062
ALP (IU/L)	80 ± 20	82 ± 21	77 ± 19	0.263
Albumin (g/L)	47 ± 3	47 ± 3	47 ± 3	0.331
GGT (IU/L)	72 ± 56	67 ± 61	81 ± 45	0.104
AST (IU/L)	52 ± 31	46 ± 34	62 ± 23	0.003*
Platelet count (× 10^9^/L)	210 ± 62	227 ± 48	183 ± 73	0.017*
Cholesterol (mmol/L)	5.14 ± 1.38	5.29 ± 1.04	4.86 ± 1.87	0.559
HDL (mmol/L)	1.21 ± 0.35	1.25 ± 0.37	1.13 ± 0.32	0.252
LDL (mmol/L)	3.42 ± 1.00	3.52 ± 0.90	3.23 ± 1.20	0.240
Triglycerides (mmol/L)	2.15 ± 1.04	1.82 ± 0.79	2.13 ± 1.12	0.007*

Values shown as mean ± standard deviations, unless stated otherwise. *Significant differences. Shapiro-Wilk test for normality used. *p < 0.05. *DM*, diabetes mellitus; *ALT*, alanine aminotransferase; *AST*, aspartate aminotransferase; *BMI*, body mass index; FIB4, Fibrosis-4; *GGT*, gamma glutamyl transferase; *ALP*, alkaline phosphatase; *HDL*, high-density lipoprotein; *LDL*, low-density lipoprotein

### Comparison of fat fraction

**Fig. 3:**
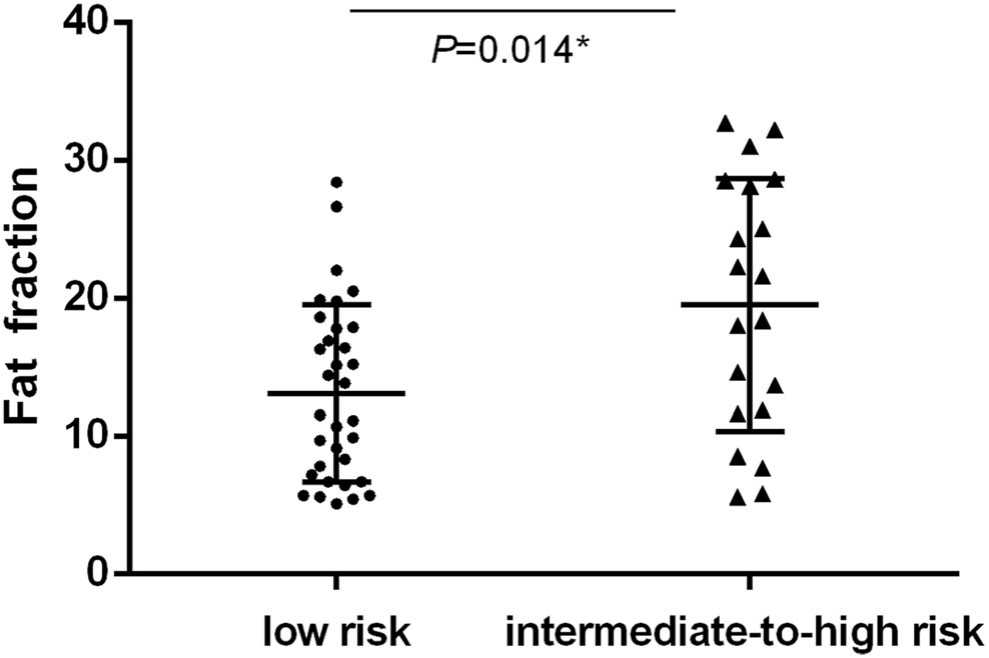
Liver fat fraction comparison between low and intermediate-to-high risk of advanced fibrosis

The liver fat fraction estimated via HISTO was compared between patients with a low risk and intermediate-to-high risk of advanced fibrosis (Fig. 3). The intermediate-to-high-risk group demonstrated an increased amount of fat compared with the low-risk group (19.31 vs. 12.98%, *p* = 0.014).

### Comparison of histogram- and texture-extracted parameters of T1 map

**Fig. 4:**
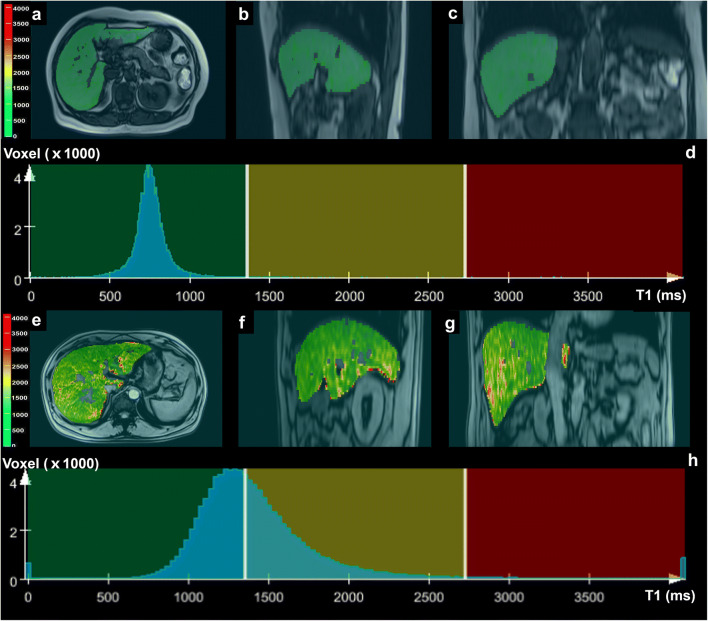
T1-map from the liver of a 40-year-old female with a low risk of advanced fibrosis (**a**–**d**) and a 39-year-old-male with a high risk of advanced fibrosis (**e**–**h**). Color maps of T1 map (**a**, **b**, **c**, **e**, **f**, **g**) and histogram of T1 map (**d**, **h**)

The univariate analyses of the extracted features are shown in Table 2. Eleven histogram and texture parameters (volume, mean, SD, median, 5th percentile, 95th percentile, kurtosis diff-entropy, diff-variance, contrast, and entropy) showed significant differences between the low-risk group and intermediate-to-high-risk group (all *p* < 0.05, respectively). Representative histogram and texture analysis on the T1 maps of two patients are shown in Fig. 4**.**

**Table 2:** Histogram and texture analysis-based extracted parameters of T1 map between low and intermediate-to-high risk of advanced fibrosis

Parameters	Low risk of advanced fibrosis (*n* = 33)	Intermediate-to-high risk of advanced fibrosis (*n* = 20)	*p*
Volume (cm^3^)	1075.88 ± 318.75	1485.41 ± 531.52	< 0.001*
T1 mean (ms)	954.25 ± 278.17	1265.9 ± 351.06	0.001*
SD (ms)	290.09 ± 178.64	481.60 ± 244.44	0.005*
Median (ms)	915.83 ± 257.46	1176.91 ± 315.55	0.002*
5th percentile (ms)	627.51 ± 176.85	744.78 ± 152.82	0.030*
95th percentile (ms)	1359.80 ± 605.78	2079.55 ± 967.95	0.004*
Kurtosis	34.58 ± 24.71	17.84 ± 16.10	0.010*
Diff-entropy	1.27 ± 0.27	1.67 ± 0.35	< 0.001*
Diff-variance	2.37 ± 2.42	4.56 ± 3.67	< 0.001*
Contrast	6.01 ± 6.88	13.27 ± 11.08	< 0.001*
Entropy	2.07 ± 0.43	2.63 ± 0.44	< 0.001*
Skewness	3.31 ± 2.52	2.49 ± 1.61	0.117

Data are expressed as mean ± standard deviations. *p < 0.05

The significantly different histogram and texture parameters showed diagnostic performances with AUCs in ROC analyses ranging from 0.679 to 0.837 when used as a single parameter in a model, except for kurtosis (Table 3). Diff-entropy had the highest AUC of 0.837 (95% CI 0.73–0.95, sensitivity 84.6%, specificity 77.8%). Binary logistic regression analysis was run on the eleven parameters with a forward stepwise selection procedure; the optimal combination included the median, 5th percentile, and diff-entropy (ESM 1), yielding an AUC of 0.902 (95% CI 0.788–0.966, sensitivity of 80.0%, specificity of 90.9%) (Table 3). We defined this as the first multivariate model, which was assessed with the goodness-of-fit Hosmer-Lemeshow test (*p* = 0.731). A DeLong test showed that AUC of the multivariate model was significantly higher than that of individual parameters (volume, mean, SD, median, 5th percentile, 95th percentile, diff-variance, contrast, and kurtosis, all *p* < 0.05) (Table 4), except for diff-entropy (*p* = 0.071) and entropy (*p* = 0.050).

**Table 3:** ROC analyses of liver volumetric histogram- and texture-extracted parameters in differentiating low risk from intermediate-to-high risk of advanced fibrosis

Parameters	AUC	95% confidence interval	Cutoff value	Youden index	Sensitivity (%)	Specificity (%)	*p*
Volume	0.780	0.64–0.91	1289.85	0.58	70.0	87.9	0.001*
T1 Mean	0.771	0.64–0.91	987.52	0.51	84.6	66.7	0.001*
SD	0.733	0.60–0.88	331.63	0.44	76.9	66.7	0.005*
Median	0.757	0.62–0.90	914.50	0.51	84.6	66.7	0.002*
5th percentile	0.679	0.52–0.83	721.5	0.47	69.2	77.8	0.030*
95th percentile	0.736	0.59–0.83	1541.5	0.51	76.9	74.1	0.004*
Kurtosis	0.289	0.15–0.43	0.48	0.04	100	3.7	0.010*
Diff-entropy	0.837	0.73–0.95	1.40	0.62	84.6	77.8	0.000*
Diff-variance	0.807	0.69–0.93	2.05	0.51	84.6	66.7	0.000*
Contrast	0.793	0.67–0.92	5.29	0.55	84.6	70.4	0.000*
Entropy	0.821	0.71–0.94	2.33	0.55	84.6	70.4	0.000*
Multivariate model	0.902	0.79–0.97	0.46	0.71	80.0	90.9	0.000*

*AUC*, area under receiver operating characteristic curve. *p < 0.05. Cutoff values were established by calculating the maximal Youden index (Youden index = sensitivity + specificity −1)

**Table 4:** Comparison of AUC between multivariate models and each parameter or blood fibrosis test

Parameters	Difference between AUC (95%CI)	Z	*p*
Volume	0.121 (−0.022–0.264)	1.663	0.0063*
T1 mean	0.130 (0.028–0.233)	2.486	0.0129*
SD	0.168 (0.066–0.270)	3.226	0.0013*
Median	0.145 (0.034–0.255)	2.560	0.0105*
5th percentile	0.402 (0.317–0.486)	9.321	< 0.0001*
95th percentile	0.402 (0.317–0.486)	9.321	< 0.0001*
Kurtosis	0.190 (0.065–0.315)	2.993	0.0028*
Diff-entropy	0.065 (− 0.006–0.136)	1.807	0.0707
Diff-variance	0.095 (0.016–0.173)	2.362	0.0182*
Contrast	0.108 (0.022–0.195)	2.449	0.0143*
Entropy	0.079 (− 0.000–0.158)	1.958	0.0503
FIB-4	0.247 (0.068–0.426)	2.709	0.0067*
NSF	0.223 (0.037–0.408)	2.353	0.0186*
LSM	0.098 (0.0141–0.183)	2.286	0.0222*

*AUC*, area under receiver operating characteristic curve. *p < 0.05. *NSF*, Nonalcoholic Fatty Liver Disease Fibrosis Score (NFS); *FIB-4*, fibrosis-4 index; *LSM*, liver stiffness measurement

When adding significantly different clinical parameters (DM proportion, AST, platelet count, and triglycerides) and MRS_FF to the first multivariate model, diagnostic performance showed no better than that of the first multivariate model (*p* = 0.974, *Z* = 0.032). The optimal combination included platelet count, 5th percentile, and diff-entropy yielding an AUC of 0.900 (95% CI 0.786–0.965, sensitivity of 85.0%, specificity of 87.9%). So we choose the first multivariate model with three parameters (median, 5th percentile, and diff-entropy) for further analysis.

### Comparing the noninvasive fibrosis test with multivariate model for differentiating the intermediate-to-high-risk group from the low-risk group

**Fig. 5:**
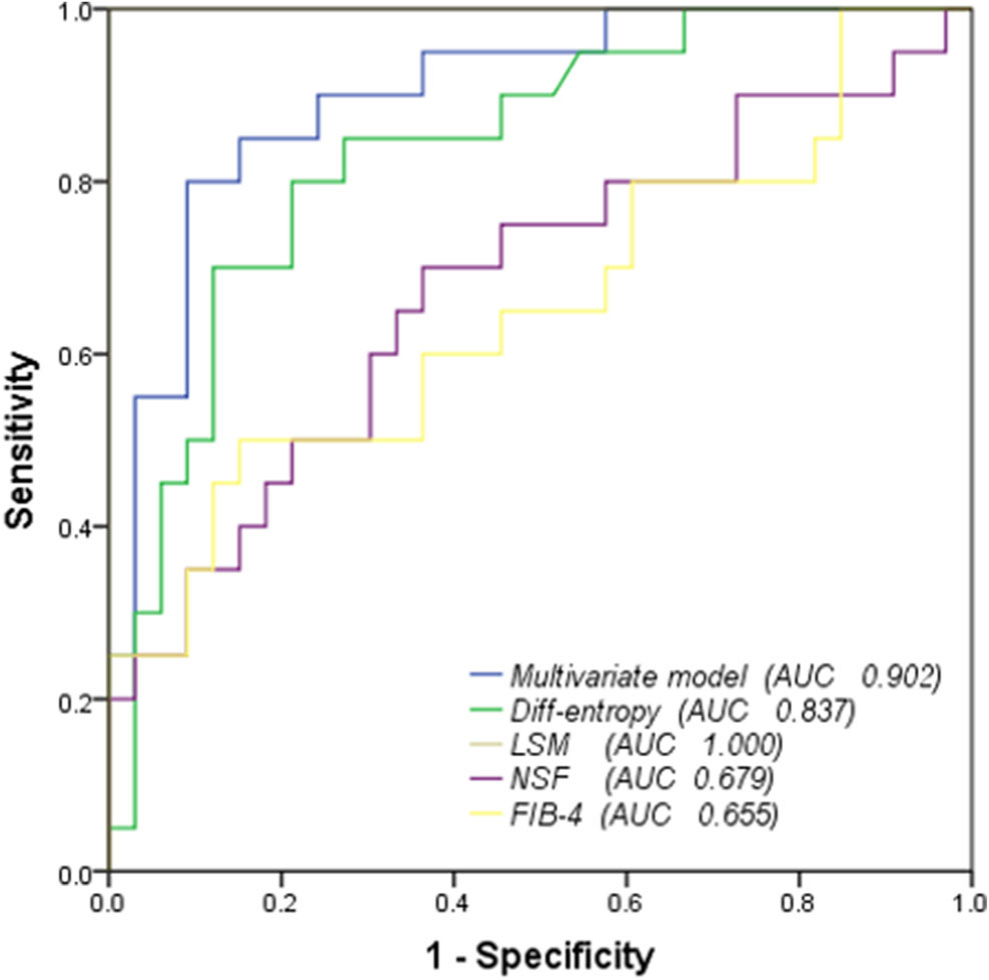
Receiver operating characteristic (ROC) curve for the differentiation between a low and intermediate-to-high risk of advanced fibrosis. The multivariate model was derived from the logistic regression analysis of the histogram and texture parameters. NSF, Nonalcoholic Fatty Liver Disease Fibrosis Score; FIB-4, Fibrosis-4 Index; LSM, liver stiffness measurement

The diagnostic performance of multivariate model was compared with noninvasive fibrosis tests including NFS, FIB-4, and LSM, respectively (Fig. 5, Table 4). The multivariate model indicated significantly better performances when comparing with NFS (*p* = 0.0186, *Z* = 2.353) and FIB-4 (*p* = 0.0067, *Z* = 2.709). However, as only a slight difference of 0.098 of AUC was observed between multivariate model and LSM (*p* = 0.022, *Z* = 2.286), the diagnostic performance of multivariate seemed no better than LSM.

### Intra- and inter-observer variability and expenditure of time

The ICC analysis for inter- and intra-observer variability is summarized in Tables 5, respectively. Intra-observer ICCs ranged from 0.827 (0.613–0.958) for diff-entropy to 0.992 (0.969–0.998) for entropy. Inter-observer ICCs ranged from 0.808 (0.597–0.951) for diff-variance to 0.984 (0.937–0.996) for median.

**Table 5:** Intra- and inter-observer variability for whole-liver histogram and texture parameters

Parameters	Intra-observer ICC (95% CI)	Inter-observer ICC (95% CI)
Volume	0.977 (0.927–0.993)	0.910 (0.683–0.977)
T1 Mean	0.834 (0.693–0.958)	0.971 (0.967–0.988)
SD	0.859 (0.745–0.956)	0.877 (0.584–0.968)
Median	0.974 (0.918–0.992)	0.984 (0.937–0996)
5th percentile	0.828 (0.545–0.957)	0.864 (0.548–0.964)
95th percentile	0.930 (0.753–0.984)	0.878 (0.687–0.968)
Skewness	0.945 (0.792–0.987)	0.910 (0.683–0.977)
Kurtosis	0.828 (0.615–0.978)	0.826 (0.616–0.953)
Diff-entropy	0.827 (0.613–0.958)	0.887 (0.672–0.976)
Diff-variance	0.962 (0.880–0.989)	0.808 (0.597–0.951)
Contrast	0.979 (0.918–0.995)	0.837 (0.643–0.978)
Entropy	0.992 (0.969–0.998)	0.945 (0.794–0.986)

*ICC*, intraclass correlation coefficient; *CI*, confidence interval

ESM 2 reports mean times required by each reader for drawing seed points and total time for four steps. For both readers, the total four steps required significantly more time than drawing seed points (both *p* < 0.05); manual adjustments are needed nearly in all cases. Finally, by comparing time between readers, no significant difference was found in the time of drawing seed points and total time (*p* > 0.05), though the more experienced reader 2 had a slightly shorter time for each case (399 ± 212 s vs 433 ± 252 s in seed point drawing and 794 ± 481 s vs 678 ± 367 s in total time).

### Inter-examination repeatability

Inter-examination ICCs for volume, mean, SD, median, 5th percentile, 95th percentile, skewness kurtosis, diff-entropy, diff-variance, contrast, and entropy were 0.970, 0.994, 0.997, 0.974, 0.960, 0.999, 0.947, 0.877, 0.981, 0.991, 0.990, and 0.982 respectively (ESM 3).

## Discussion

In this study, we assessed the potential of whole-liver histogram and texture analysis of T1 map in stratifying the risk of advanced fibrosis in the fatty liver. In a selected participant cohort with suspected NAFLD, a 2-step approach, combing NFS/FIB-4 and liver stiffness, was performed to distinguish individuals as being in the intermediate-to-high risk of advanced fibrosis group and low-risk group. The NFS [20] and FIB-4 [21, 22] index have been externally validated in populations of different ethnicities with consistent results. LSM is clinically useful to monitor the severity of hepatic fibrosis and is recommended in the current guidelines on management of NAFLD [4, 23].

The presence of advanced fibrosis in NAFLD identifies patients in need of in-depth hepatological investigation, and it is an independent predictor of liver-related mortality [2]. The monitoring of fibrosis progression is necessary at variable time intervals. The need for a noninvasive alternative to liver biopsy has led to the extensive investigation of predictive methods, including the serum fibrosis test, elastography by US and MRI, and other imaging methods. Although MR-based elastography techniques have demonstrated the best performance for staging hepatic fibrosis in patients with NAFLD [24], a prospective acquisition with dedicated hardware is required.

Histogram and texture analyses as statistical tools have been increasingly used in staging liver fibrosis in chronic liver disease [25, 26]. Here, we extracted histogram and texture parameters on the T1 map for the following reasons: (*i*) Its implementation does not require any additional hardware or contrast agent and could be applied in almost every MR machine without any extra cost; (*ii*) NAFLD progression includes various pathological and micro-structural changes that might not be detectable by the human eye, like steatosis, fibrosis, inflammation, and iron deposition, which promotes fibrosis progression. All the substances above will affect the T1 values. We assumed that the texture from the T1 map might provide comprehensive and important information concerning liver fibrosis.

In our study, the histogram-based (volume, mean, SD, median, 5th percentile, and 95th percentile) and texture-based (diff-entropy, diff-variance, contrast, and entropy) parameters all demonstrated a good diagnostic performance, with high sensitivity ranging from 69.2 to 84.6% and specificity from 66.7 to 87.9%, except for kurtosis. All parameters showed excellent inter- and intra-observer agreement. Consistent with other studies [26–29], the most promising parameters of our study are diff-entropy, entropy, and diff-variance, achieving an AUC value greater than 0.8 (*p* < 0.001) in differentiating intermediate-to-high-risk advanced fibrosis from low risk.

Although the texture of T1map was rarely studied in the abdomen, animal experiments have found that the texture parameters of T1 map can be used for detection of hepatic fibrosis [26]; studies in patients with NAFLD have shown that entropy on T1-weighted images can help measure hepatic fibrosis [27]; Fujimoto et al [28] have reported that entropy of ADC map correlated with pathologic fibrosis stage in chronic hepatitis C; Yang et al [29] have demonstrated that histogram analysis of susceptibility-weighted imaging, particularly the variance, can be used for predicting advanced liver fibrosis. Histopathologically, the early accumulation of fat within the liver tends to be diffuse and uneven distributed in NAFLD [30]; the fatty acids (secreted from the fat) can contribute to the formation of lipotoxic species, oxidant stress, and inflammasome activation. These processes are responsible for accumulation of excess ECM, which might lead to advanced fibrosis [31]. As the distribution of those substances (fat, inflammation, ECM, fibrosis) becomes more heterogeneous, the distribution of T1 value therefore becomes more random, and the entropy increases. Variance also increases when T1 map becomes more heterogeneous. In total, the observation of increased diff-entropy, entropy, and diff-variance of T1 map at volumetric texture analysis may therefore be a potential biomarker that reflects increased heterogeneity of hepatic parenchyma in diffuse liver disease. Histogram-based parameters (volume, mean, SD, median, 5th percentile, and 95th percentile) could provide insight into the distribution of T1 values over the entire liver. All these parameters demonstrated a good diagnostic performance in our study.

In addition, texture analysis performed in other organs indicated that the combination of parameters might increase the clinical diagnostic performance [32–35]. Our results indicated that the combination of histogram and texture parameters using binary logistic regression analysis showed a better diagnostic performance compared with most individual parameters or conventional blood fibrosis tests (NFS and FIB-4)**.** In order to verify whether clinical parameters could improve the diagnostic efficiency, we added all significantly different clinical indicators to the multivariate model and found that there was no difference in AUCs (0.902 vs 0.900), indicating that the clinical parameters do not offer added value to the model.

- There is a small set of previous studies [12, 27] focusing in a similar fashion on a homogeneous participant cohort with NAFLD. Notably, the parameters in those studies were extracted using a single circular ROI placed on a slice, which have caused the loss of heterogeneity information of the whole liver. In the current study, we utilized the whole volume analysis on the entire liver, which could extract more comprehensive information on the whole liver. The influences of the hepatic vessels and intrahepatic bile duct were avoided by excluding them during the semi-automatic segmentation processing. We acquired the 3D T1 map by using the variable flip angle method [32] in a single breath-hold period. B1 correction was applied on the T1 map to ensure the accuracy of the T1 estimation. We found the repeatability of T1mapping after repositioning to be excellent, as the inter-examination ICCs were all > 0.85.

Our study had several limitations. First, the patient population was relatively small (*n* = 53), so the diagnostic criteria proposed in our study should be validated in another cohort. Second, due to the study design, liver biopsy was not possible, we instead used MRS-FF as a noninvasive gold standard of steatosis, and a 2-step approach to evaluate advanced fibrosis risk in NAFLD patients, which have been proposed in the guidelines of the European Association for the Study of the Liver (EASL) [4] and confirmed by several published studies using histology as the reference standard [36, 37]. Third, the current semi-automatic segmentation-based analysis is time-consuming. It took an average of ~ 13 min for reader 1 who is unexperienced and ~ 11 min for reader 2 who is more experienced per case for the whole process in our study; further texture analysis from automatic liver segmentation and vessel exclusion might help; fourth, we only analyzed T1 maps on 1.5T scanner. The accuracy of histogram and texture parameters using 3T scanner or another sequence remains unknown, and needs further study. Finally, our retrospective study allowed the evaluation of the potential role of whole-liver texture analysis on T1 maps for the risk stratification of advanced fibrosis in NAFLD; however, selection bias can occur in retrospective cohort studies as the patient outcome is known at baseline at the time the study is initiated. To validate our findings, a randomized prospective study should be performed.

In conclusion, whole volume histogram and texture analysis on T1 maps has the potential to discriminate between low-risk and intermediate-to-high-risk advanced fibrosis. The combination of significant texture parameters yielded a better performance for the risk stratification of advanced fibrosis in NAFLD, and clinical parameters offers no added value. These results warrant further studies with a larger patient population to confirm our findings.

## Electronic supplementary material

ESM 1 (DOCX 19 kb)

## Abbreviations

ALP: Alkaline phosphatase
ALT: Alanine aminotransferase
AST: Aspartate aminotransferase
AUC: Area under the receiver operating characteristic curve
BMI: Body mass index
CI: Confidence interval
DM: Diabetes mellitus
ECM: Extracellular matrix
FF: Fat fraction
FIB-4: Fibrosis-4 index
GGT: Gamma glutamyl transferase
HDL: High-density lipoprotein
LDL: Low-density lipoprotein
LSM: Liver stiffness measurement
NAFLD: Nonalcoholic fatty liver disease
NSF: Nonalcoholic fatty liver disease fibrosis score
ROC: Receiver operating characteristic
VFA: Variable flip angle
VOI: Volume of interest
