# Supplementary material for: Whole-liver histogram and texture analysis on T1 maps improves the risk stratification of advanced fibrosis in NAFLD
Source: Eur Radiol. 2020 Sep 8;31(3):1748–59. doi: 10.1007/s00330-020-07235-4 (PMC7880972; doi:10.1007/s00330-020-07235-4)
Supplement: Supplementary file 1 — (DOCX 19 kb) [file 330_2020_7235_MOESM1_ESM.docx]

**ESM 1**. The multivariate model for differentiating a low risk from an intermediate-to-high risk of advanced fibrosis in NAFLD patients.

| Multivariate model | β | S.E. | *P* | OR |
| --- | --- | --- | --- | --- |
| Median | -0.014 | 0.006 | 0.022 | 0.986 |
| 5th percentile | 0.006 | 0.004 | 0.148 | 1.016 |
| Diff-entropy | 15.891 | 5.426 | 0.003 | 7.96*10^6 |
| Constant | -13.241 | 3.675 | 0.000 | 0.000 |

Note; β, Partial regression coefficient; OR, odds ratio; SE, standard error.

ESM2. Average time (in seconds) used for seed-point drawing and total time for both readers

|  | Seed-point drawing | Total time | *P* |
| --- | --- | --- | --- |
| Reader 1 | 433±252 | 794±481 | 0.002* |
| Reader 2 | 399±212 | 678±367 | 0.002* |
|  | *P*>0.05 | *P*>0.05 |  |

Data are expressed as mean ± standard deviations. **P<0.05.*

ESM3. Inter-examination repeatability of T1 maps between the three acquisitions

| Parameters | Inter-examination  ICC [95% CI] |
| --- | --- |
| Volume | 0.970 [0.871-0.997] |
| T1 Mean | 0.994 [0.975-0.999] |
| SD | 0.997 [0.986-0.999] |
| Median | 0.974 [0.891-0.997] |
| 5th percentile | 0.960 [0.824-0.995] |
| 95th percentile | 0.999[0.994-0.999] |
| Skewness | 0.947 [0.789-0.993] |
| Kurtosis | 0.877 [0.575-0.985] |
| Diff-entropy | 0.981 [0.913-0.998] |
| Diff-variance | 0.991 [0.960-0.999] |
| Contrast | 0.990 [0.957-0.998] |
| Entropy | 0.982 [0.916-0.997] |

ICC, intraclass correlation coefficient; CI, confidence interval
